# Substrate Specificity of GSDA Revealed by Cocrystal Structures and Binding Studies

**DOI:** 10.3390/ijms232314976

**Published:** 2022-11-29

**Authors:** Qian Jia, Jinbing Zhang, Hui Zeng, Jing Tang, Nan Xiao, Shangfang Gao, Huanxi Li, Wei Xie

**Affiliations:** MOE Key Laboratory of Gene Function and Regulation, School of Life Sciences, Sun Yat-Sen University, Guangzhou 510006, China

**Keywords:** guanosine deaminase, cocrystal structure, conformational diversity, purine metabolism

## Abstract

In plants, guanosine deaminase (GSDA) catalyzes the deamination of guanosine for nitrogen recycling and re-utilization. We previously solved crystal structures of GSDA from *Arabidopsis thaliana* (AtGSDA) and identified several novel substrates for this enzyme, but the structural basis of the enzyme activation/inhibition is poorly understood. Here, we continued to solve 8 medium-to-high resolution (1.85–2.60 Å) cocrystal structures, which involved AtGSDA and its variants bound by a few ligands, and investigated their binding modes through structural studies and thermal shift analysis. Besides the lack of a 2-amino group of these guanosine derivatives, we discovered that AtGSDA’s inactivity was due to the its inability to seclude its active site. Furthermore, the C-termini of the enzyme displayed conformational diversities under certain circumstances. The lack of functional amino groups or poor interactions/geometries of the ligands at the active sites to meet the precise binding and activation requirements for deamination both contributed to AtGSDA’s inactivity toward the ligands. Altogether, our combined structural and biochemical studies provide insight into GSDA.

## 1. Introduction

As essential components of biomolecules, purines are raw materials for nucleic acid synthesis, and the precursors of primary products in numerous biosynthetic processes, including sucrose, polysaccharides, and phospholipids, etc. In addition, they also provide energy for living organisms and regulate key metabolic processes [1]. Therefore, the biosynthesis and metabolism of nucleotides are of great significance to the growth and development of all organisms [2,3].

Purine metabolism refers to converting purine nucleotides to carbon dioxide and ammonia, which involves their sequential dephosphorylation, deamination and glycosidic bond-cleavage reactions to produce the crucial intermediate xanthine [3]. The dephosphorylation produces guanosine (Gua), whose subsequent degradation is conducted through two possible metabolic pathways. The first is the guanosine deaminase (GSDA)-mediated conversion to form xanthosine (Xan), which is then hydrolyzed to give xanthine. The other involves the hydrolysis of Gua to guanine (G), followed by its deamination to xanthine under the action of guanine deaminase (GDA) [4,5,6]. Both deaminases are zinc-dependent enzymes, which rely on a catalytic water molecule to attack the amino group-connecting C2 atom to proceed with the catalysis. Despite their strong homology, the deaminases GSDA and GDA show strict specificity for their respective substrates (Gua and G). Moreover, GSDA has been identified almost exclusively in plants, while GDA exists in other eukaryotes and prokaryotes [5,6].

Unlike animals, plants completely catabolize purine and pyrimidine ribonucleosides for amino acid biosynthesis [7,8]. GSDA is responsible for the initial step of rN catabolism. Several phenotypes of *gsda* mutants have been reported including delayed germination, yellowing, etc. [9]. The abnormal phenotypes are possibly due to the accumulation of toxic ribonucleosides or the lack of degradation products. For example, in plant seeds and seedlings lacking *gsda*, the substrates Gua and GTP accumulated, and the latter seriously inhibited the activity of plant AMP deaminase.

Previously, we reported crystal structures of GSDA from *Arabidopsis thaliana* (AtGSDA) in the apo- and substrate/product-bound forms and proposed a catalytic model [10]. The dimerization interface of the enzyme is mainly formed by the α2, α3, α4, and α6 helices. The zinc ions are tightly bound to the active site, coordinated by the completely conserved residues His80, Cys113, Cys110, and the key water molecule. In addition, Glu82 acts as the general base to extract a proton from the catalytic water molecule, preparing the latter for the nucleophilic attack. During deamination, both the enzyme and the substrate undergo conformational changes. Specifically, the essential C-terminal loops of the enzyme would re-cap the active site by forming the key contact with N7 of Gua via the side chain of Tyr185. Meanwhile, the purine ring would rotate a 45°-angle after the attack by the hydroxyl group, but the ribose basically remains at the same location. Despite its reported strict specificity toward Gua only, we managed to identify that 2′-O-methylguanosine (2′-O-mG) and N^2^-methylguanosine (N^2^-mG) could be employed as reasonable substrates of AtGSDA by testing a series of Gua derivatives, but the structural basis is unclear. Here, we investigated the binding patterns of the ligands by determining their cocrystal structures. We discovered that most Gua derivatives failed to activate GSDA’s activity. Due to the lack of proper functional groups in these ligands, their non-optimal binding geometries prevent the enzymatic reaction from happening. Additionally, the flexible C-terminus of the enzyme may extend to the solvent and make it unable to seal the active site, so the deamination reaction would not occur either.

## 2. Results

### 2.1. Binding Modes of Different Substrates

In order to investigate the substrate specificity problems of AtGSDA, we cocrystallized or soaked AtGSDA with quite a few ligands that would either react with or inhibit the enzyme and solved their complex structures (Supplementary Table S1). For each ligand, we collected diffraction datasets from at least two independently grown cocrystals to ensure the structural accuracies and consistencies. All the cocrystal structures showed well-covered density; they all closely resemble the substrate- or the product-bound AtGSDA structures, and therefore we will mainly discuss their structural details hereinafter according to their action profiles.

**Figure 1:**
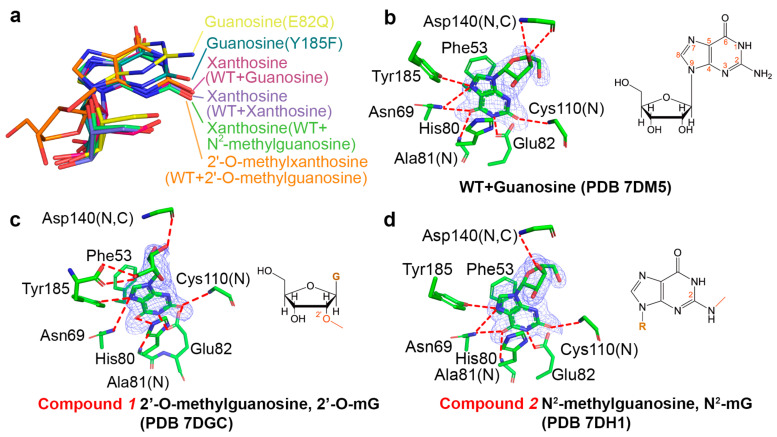
The binding modes of various substrates. (**a**) The superimposition of different ligands after the reactions with the WT enzyme or Y185F. The reaction products for guanosine, 2′-O-methylguanosine, N^2^-methylguanosine all nearly coincide with xanthosine. (**b**–**d**) The omit electron density maps showing the interactions with the ligands guanosine (**b**), 2′-O-methylguanosine (**c**), N^2^-methylguanosine (**d**). The omit-maps are contoured at 3σ. The chemical structures of the ligands are drawn with their unique parts in red. G: guanine; R: ribose.

AtGSDA acts on three substrates [10]: the natural substrate Gua, 2′-O-mG and N^2^-mG (compounds **1**–**2** in Figure 1). As a reference, we first cocrystallized the natural substrate Gua with the WT enzyme. The quick soaking process (within 2 min) resulted in the complete deamination. Besides the rapid conversion of the natural substrate, the other two substrates were almost completely converted as well, because their planar purine rings almost coincided with that of Xan bound by AtGSDA (Figure 1a). Among these, the AtGSDA structure of Xan converted from Gua (PDB 7DM5, Figure 1b) produced by their direct binding was indistinguishable to that of the AtGSDA-Xan complex reported previously (PDB 7DCA) [10]. The RMSD value between the two structures was ~0.1 Å, indicating close structural resemblance.

Interestingly, 2′-O-mG was different from the canonical Gua binding mode in that its ribose ring flips ~180°, which brought the 5′-hydroxyl to the vicinity of Tyr185 and formed hydrogen bonds with its carboxylate group; however, the ribose lost the contact with the mainchain of Asn140 instead (Figure 1c). In comparison, the enzymatic reactivity toward N^2^-mG was relatively weak [10], because the extra methyl group remarkably reduced the enzymatic activity of AtGSDA. However, the density map showed that the reaction nevertheless attained the product stage, probably due to the long incubation period under a high enzyme concentration (Figure 1d).

**Figure 2:**
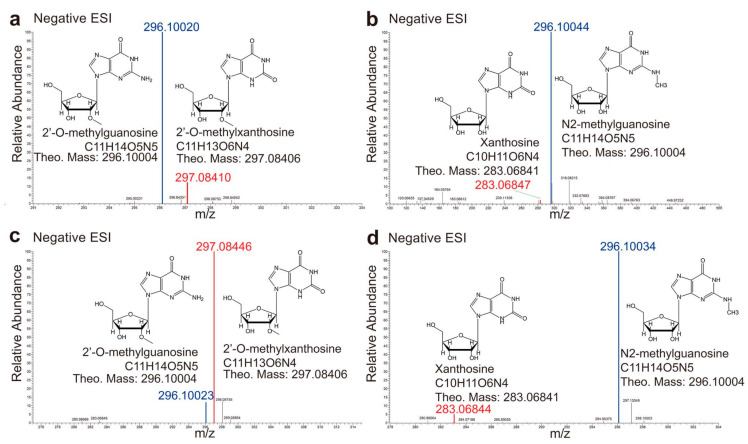
The deamination activity tests of AtGSDA and variants, analyzed by the Orbitrap high-definition mass spectrometry. (**a**,**b**) The 2-h deamination product by WT-AtGSDA incubation with 2′-O-mG (**a**) and with N^2^-mG (**b**), respectively. (**c**,**d**) The 24-h deamination product by WT-AtGSDA incubation with 2′-O-mG (**c**), and with N^2^-mG (**d**), respectively. The blue and red lines indicate the substrates and the products, respectively.

To confirm the completion extents of the deamination process, the deamination reactions were carried out at an enzyme/substrate molar ratio of 1:100, followed by detection of the product on a high-resolution Orbitrap high-definition mass spectrometer at various time points. The results indicated that the starting material Gua in a reaction would completely disappear in 2 h (Supplementary Figure S1a), while only ~15% 2′-O-mG or very little N^2^-mG was converted into the product (Figure 2a,b). By the 24-h time point, the 2′-O-mG reaction would almost finish while the majority of N^2^-mG was still in the substrate form (Figure 2c,d). In contrast, E82Q did not produce any products when incubated with 2′-O-mG or N^2^-mG under similar assay conditions (Supplementary Figure S1b–d).

### 2.2. Binding Modes of Different Inhibitors

The inhibitor compounds (**3**–**5**) normally lacked one or two functional groups (including the key 2-amino group) for hydrogen-bond interactions. Nevertheless, they adopted the nearly perfect substrate-binding geometries in the structures, and thus acted as competitive inhibitors.

**Figure 3:**
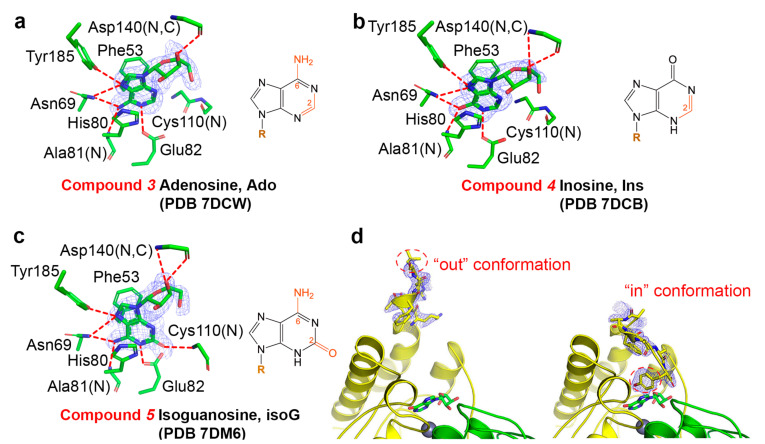
The inhibition modes of compounds **3**–**5.** (**a**–**c**) The omit electron density maps showing the interactions with the inhibitor adenosine (**a**), inosine (**b**), and isoguanosine (**c**). The maps are contoured at 3σ and only the more tightly bound ligands are shown. The chemical structures of the ligands are drawn with their unique parts in red. G: guanine; R: ribose. (**d**) The two possible conformations of the C-terminus observed in the WT-inosine complex (PDB 7DCB). The two chains are shown in ribbon representation and colored green and yellow, respectively. The spheres indicate the catalytic zinc ions. Note that in the final model, the “in” conformation (also the major conformation) is modeled.

The ligand adenosine (Ado) maintained its interactions with Ala81 and Asn69 via N6 as did O2 of Gua, but the hydrogen bond with the 2-amino group by Cys107 was lost (Figure 3a, PDB 7DCW). Asn69 is conserved in GDA, a key residue for substrate anchoring and catalysis [11]. This binding mode created a distance of ~4.5 Å between C6 and the catalytic water, too far for the nucleophilic attack. Therefore, unlike adenosine deaminase (ADA), AtGSDA is not capable of deaminating Ado. Inosine (Ins) is identical to Gua except that the 2-amino group is missing (Figure 3b, PDB 7DCB); it is usually formed by ADA through its deamination reaction on Ado. The binding position was almost unchanged when compared to Gua bound by AtGSDA.

On the other hand, isoguanosine (isoG) is the isoform of Gua with the exocyclic amino and keto groups switched, and its binding mode was similar to that of Xan (Figure 3c, PDB 7DM6). Consequently, its 2-keto group provided the lone-pair electrons needed for the ligation of zinc and the original water ligand was dislodged. isoG was mimicking the product Xan although its C4 position was attached to an amino group.

Notably, in these three structures, the C-termini of the enzyme were buried into the active sites, with Tyr185 making the hydrogen bond with N7 of these compounds (named the “in” conformation), except that the C-terminus of one monomer of the Ins-bound structure showed an alternative conformation with residues Glu180-Tyr185 extending to the solvent (the “out” conformation, Figure 3d). Because this conformation only represented a minor fraction of the enzyme ensemble, we still modeled the “in” conformation in the final structure for this chain. Despite these subtle differences, the remaining parts of the two chains (including ligands) could be well superimposed. Additionally, the B-factors for the bound ligands were also reasonably close to each other, suggesting that the two chains were equally important in binding the ligands.

**Figure 4:**
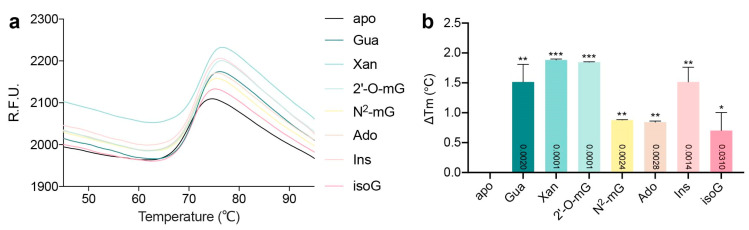
The TSA melting curves of various ligands to AtGSDA. (**a**) The melting curves of the enzyme in complex with various ligands. The horizontal axis indicates temperature while the vertical axis indicates relative fluorescence units (R.F.U.). (**b**) The comparison of the stabilizing effects brought by the ligands. ΔTm represents the changes in Tm values between the complexes and the apo-enzyme. Error bars are standard deviation (s.d.) (*n* = 3 biological replicates). Statistical evaluation with ANOVA is followed by Tukey’s post test. Probability values for pairwise comparisons to apo are shown at the respective bars. *: *p* < 0.05, **: *p* < 0.01, ***: *p* < 0.001.

To further test the binding affinities of the enzyme toward these compounds, we employed thermal shift analysis (TSA). This assay assesses the structural integrity and thermostability of a protein by measuring its “structural openness” with a temperature gradient (i.e., a heat denaturation) and uses a dye that binds the hydrophobic core of the protein. This binding upon the unfolding of the target protein during heating would produce a signal, at a temperature which is called the “melting temperature” (Tm). However, the ligands tend to stabilize the enzyme due to their mutual interactions, thus conferring enhanced thermostabilities to AtGSDA. We found that the product Xan and 2′-O-mG would boost the Tm value by nearly 2 °C, which may be related to the unusual binding mode as we described earlier (Figure 4a,b). The binding of Ins produced equal stabilization effects as did by the natural substrate. In contrast, the inhibitors Ado/isoG or the weak substrate N^2^-mG normally lacked one or two functional groups (compared to Gua) that originally made hydrogen bonding interactions with the enzyme. Consequently, they brought less stabilization effects to the enzyme once they were bound. For example, Ado and Ins lacked the 2-amino group, and a ~1.0 °C decrease in the Tm value was observed when compared to Gua.

### 2.3. Binding Modes of Different Substrates by the Catalytically Impaired Mutants

AtGSDA catalysis needs two key sub-processes: the activation of water and re-capping of the active site, which proceed via the two critical residues Glu82 and Tyr185, respectively. We already cocrystalized the E82Q-Gua (PDB 7DC9), WT-Gua (PDB 7DM5) and WT-2′-O-mG (PDB 7DGC) complexes, respectively. Here, we solved the structure of E82Q bound by 2′-O-mG (PDB 7W1Q), as well as that of Y185F bound by Gua (PDB 7DQN), in order to further understand the activation mechanism of the enzyme.

**Figure 5:**
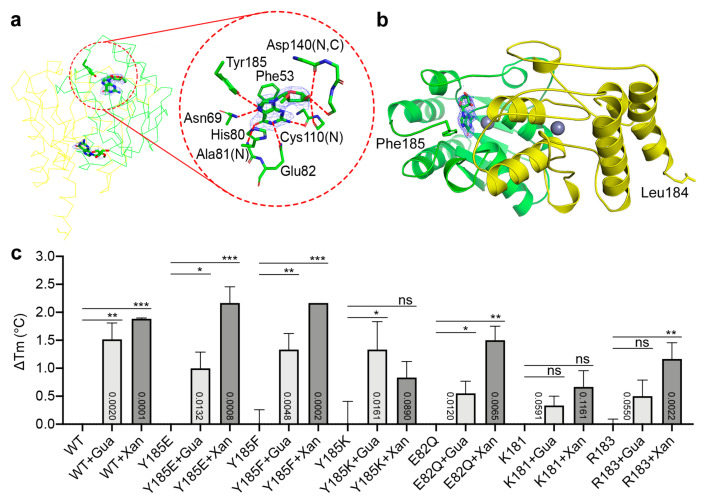
Binding modes of different substrates by the catalytically impaired mutants. (**a**) The E82Q-2′-O-methylguanosine complex structure (PDB 7W1Q). Shown on the left is the AtGSDA dimer represented in the backbone traces. The ligands and the electron density are shown. On the right is the closeup view of the interaction network of the 2′-O-methylguanosine, which resembles the substrate-binding mode, including the interactions on the ribose. (**b**) The Y185F mutant in complex with the substrate (PDB 7DQN). The last residues of the two chains are shown in sticks and labeled. (**c**) The guanosine and xanthosine binding capabilities of the mutants. Error bars are s.d. (*n* = 3 biological replicates). Statistical evaluation with ANOVA is followed by Tukey’s post test. Probability values for pairwise comparisons to the group of apo-protein respectively, are shown at the respective bars. *: *p* < 0.05, **: *p* < 0.01, ***: *p* < 0.001. ns: no significance. The omit maps are contoured at 3σ.

The E82Q-2′-O-mG structure resembled that of E82Q-Gua, and the guanine rings coincided well, suggesting the non-reactive nature (Figure 5a, PDBs 7W1Q and 7DC9). The two catalytic water molecules (one per monomer) still existed, forming the ligands for the zinc ions. The distances between the water molecules and their respective C2 atoms were 2.82 and 2.88 Å, respectively, longer than that in the apo-enzyme (2.62 Å). The density of the Gua molecule bound by one chain (monomer A) was much clearer than the one bound by the other chain (monomer B), and the latter showed poorly defined density for the sugar ring. Moreover, while the C-terminus of monomer A adopted the “in” conformation with the formation of the 2′-O-mG/N7-Tyr185/OH interaction, its counterpart in monomer B adopted the “out” conformation, a state similar to that of the minor conformer in the Ins-bound enzyme or that of the apo-enzyme (one monomer with the intact C-terminus forms loops and points out) (PDBs 7DCB and 7DBF). Their respective subunits have similar temperature factors of 31.0 and 31.8 Å^2^, respectively. In contrast, the temperature factors of the two bound ligands showed relatively larger differences, which were 26.6 and 30.3 Å^2^, respectively (0.8 vs. 2.7 Å^2^). The ligand difference in temperature factors were also observed in other cocrystal structures as well [10], while their protein chains were similar. Considering the poor catalytic capability of E82Q, we speculated that the unstable binding of ligands (as suggested by the high temperature factor) and the “out” conformer of this particular subunit (B) were correlated, and the inactivity of AtGSDA probably prompted the “opening” of the active site and the pre-mature release of the bound substrate molecule. Besides the similar orientation of the purine ring, the binding mode of the sugar ring of 2′-O-mG by E82Q also took after that of Gua (i.e., the canonical mode), but was distinct from 2′-O-methylxanthosine (2′-O-mX) bound by the WT (PDB 7DGC). In the latter structure, both C-termini well capped the active sites of the WT enzyme, while chain B of E82Q was free and unfixed.

Furthermore, the Y185F-Gua structure indicated that only one Gua molecule was bound in a substrate-bound conformation but the ligand could be clearly distinguished from the non-reactive state, judged from the rotation angle of the purine ring. By contrast, no Gua substrate was bound by the other chain (Figure 5b, PDB 7DQN). This meant that the reaction was only halfway through, and therefore the substrate form (i.e., Gua) was filled in the final model. It should be noted that these cocrystal structures showed averaged results of multiple deamination reactions, as either the substrate or product could be present in individual enzyme molecules. Accordingly, the C-termini of the enzyme adopted the mixed “in/out” conformations with the last residue Phe185 in the “out” conformer missing (Figure 5b). Similar to the E82Q-2′-O-mG case (PDB 7W1Q), the catalytic water molecules were retained, and the distance between the water and C2 of the single Gua molecule was even larger (2.96 Å).

To further test the ligand binding capabilities of the mutants, we acquired the binding profiles of several AtGSDA variants in the presence of Gua or Xan (Figure 5c). These mutations involved either changes on Tyr185, or truncation at the C-terminus. We found that while WT and Y185F/E showed similar binding affinities toward the ligands, the Y185K and E82Q mutants showed differentially reduced affinities (Figure 5c). The most dramatic changes were caused by the K181 and the R183 deletions (the four- and two-residue truncation at the C-termini, respectively), both of which brought over 1.0 °C Tm reduction, suggesting even poorer sealing effects of the active sites by the truncated C-termini. The WT enzymatic reaction might generate Xan in the process, which would interfere with the true results. However, it was not difficult to tell that Xan stabilizes the enzyme more than Gua (Figure 5c).

### 2.4. Cross Activities between GSDA and GDA

**Figure 6:**
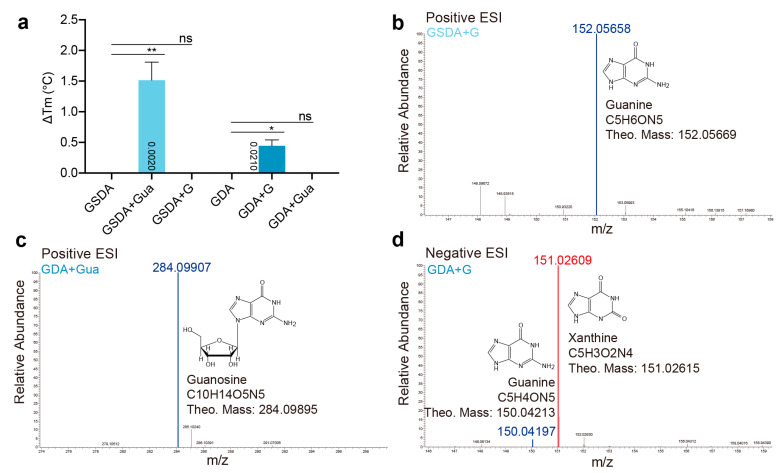
The binding and catalytic specificity of GSDA and GDA. (**a**) The guanosine and guanine binding capabilities of GSDA and GDA. Error bars are s.d. (*n* = 3 biological replicates). Statistical evaluation with ANOVA is followed by Tukey’s post test. Probability values for pairwise comparisons to the group of apo-protein respectively, are shown at the respective bars. *: *p* < 0.05, **: *p* < 0.01. ns: no significance. (**b**) The 24-h deamination reaction of AtGSDA with guanine. (**c**) The 24-h deamination reactions of GDA with guanosine. (**d**) The 2-h deamination reactions of GDA with guanine. The blue and red lines indicate the substrates and the products, respectively.

We next investigated the cross reactions of GSDA and GDA. Through TSA, we found that each enzyme displayed poor affinities toward their non-genuine substrates (Figure 6a). Accordingly, the 24-h incubation reactions of either enzyme resulted in virtually nothing in their product forms (Figure 6b,c), while GDA showed excellent deamination capability by converting almost all of the guanine substrate into xanthine in a 2-h reaction (Figure 6d). Consequently, the poor activities of the enzymes most likely resulted from the low binding affinities.

## 3. Discussion

AtGSDA displays strict substrate specificity and fails to act on most Gua derivatives, most likely due to the lack of functional groups or poor interactions/geometries of the ligands at the active sites needed to meet the precise binding and activation requirements for deamination. Here, we studied the binding modes and deamination activities of AtGSDA both through static crystal structures and biochemical studies. Although the enzyme binds the ligands similarly to that of the genuine substrate in structures, the structural analyses indicated the suboptimal distances from the catalytic water to the C2 (in the Ado case, PDB 7DCW) or improper closing of the active sites, which would lead to the activation failures of the enzyme toward these compounds. Through our crystallographic studies, we surprisingly found that the two monomers of AtGSDA bound by Ins behaved differently (PDB 7DCB), with an alternative conformation of the C-terminal tail of one subunit protruding to the solvent, although this “out” conformer only represented a small fraction. The E82Q-Gua complex (PDB 7DC9) was a more extreme case in that one of the monomers was completely extending to the solvent, and the ligand bound by this ligand showed only partial density. Lastly, the “out” conformer was also observed in the cocrystal structure of the partially active mutant Y185F, and there was only one ligand in the dimeric enzyme. Y185F had difficulties in binding to the substrate, as shown by other single mutants (Y185E/K, etc.) or truncated forms of the enzyme, while retaining the correct fold (as suggested by TSA). The Y185F conformations were reminiscent of the apo-enzyme, where it had one disordered C-terminus in one monomer while that of the other formed loops and pointed out. One thing to be noted is that the crystal structures only selected the most stable conformations and the static conformations; NMR and time-resolved crystallography would probably further help to resolve the details of the dynamic behaviors by AtGSDA.

We previously tested the activities of WT and mutants of Tyr185 toward Gua and derivatives to assess the specific influence of the N7-OH interaction to enzymatic turnover and kinetics [10]. To avoid the interference from unwanted residues at the C-terminus (the free carboxylate group of Tyr185 for the salt bridge interaction was needed), all these mutants were subcloned into an engineered pET-28a (+) vector and 6 × His tags were appended to their N-termini. WT and E82Q maintained this hydrogen bond with Xan and Gua, respectively, but the Y185F/K/E, or R183 (the two-residue truncation at the C-terminus) and K181 (the four-residue truncation at the C-terminus) deletion mutants lost it. Y185K and Y185E did not exhibit significant differences from Y185F in terms of kinetic constant [10]. Agreeably, the deamination reactions proceeded according to such an order: WT-Gua > Y185F/K/E-Gua (all three similar) > R183/K181-Gua.

**Figure 7:**
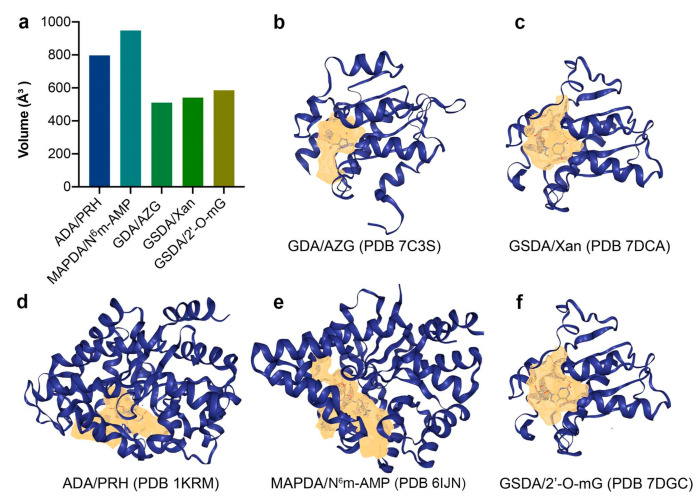
The comparison of the ligand-binding pockets of the purine-metabolic enzymes: GDA, GSDA, ADA, and MAPDA. (**a**) The volume sizes of the ligand-binding pockets. (**b**–**f**) The three-dimensional structures of GDA, GSDA, ADA, and MAPDA, with their catalytic pockets shown by the yellow mesh and the ligands shown by sticks (PDBs 7C3S, 7DCA, 7DGC, 1KRM, and 6IJN). PRH: 6-hydroxy-1,6-dihydropurine riboside, N^6^m-AMP: N^6^-methyl-AMP; AZG: 8-azaguanine; Xan: xanthosine; 2′-O-mG: 2′-O-methylguanosine.

Additionally, the flexible C-terminal region restricts the pocket size (thus conferring extra substrate selectivity by excluding inappropriate guanosine analogs). The pocket size exclusion appears to be a common mechanism adopted by enzymes involved in nucleotide metabolism with tight specificities, including N^6^m-AMP deaminase (MAPDA), ADA, GDA and GSDA, etc. [5,6,12,13,14,15]. These enzymes have distinct substrate preferences, sizes, three-dimensional structures and phylogenetic distributions, and as a result, they work on similar substrates at various levels (bases, nucleosides or nucleotides, etc.). Among these, GDA is the paralog to GSDA and both enzymes adopt the cytidine/deoxycytidine deaminase (CDA)-fold, while MAPDA and ADA are TIM-barrel enzymes catalyzing adenosine derivatives. Specially, the CDA enzymes GDA and GSDA act on guanine and guanosine, respectively; the TIM-barrel enzymes MAPDA and ADA act on adenosine and N^6^m-AMP, respectively. We therefore deduced that the substrate-binding pocket sizes of these enzymes would be different and made a calculation using DoGSiteScorer [16,17]. This grid-based tool detected the potential binding pockets of proteins based on the three-dimensional structures and calculated their volumes, hydrophobicity and enclosure parameters, etc. The volume of GDA (NE0047) was 510 Å^3^ (PDB 7C3S, only one monomer was considered), compared to the 541 Å^3^ of that of AtGSDA (PDB 7DCA for the Xan-bound complex, Figure 7a–c). Correspondingly, the depth of the former was also slightly smaller (16.2 vs. 17.1 Å, Supplementary Figure S2). This result was reasonable because GSDA binds a larger substrate molecule than GDA. Similarly, these parameters for bovine ADA were also smaller than those of MAPDA (PDBs 1KRM and 6IJN, Figure 7d,e and Figure S2). Partly for this reason, both the GSDA/guanine and GDA/guanosine pairs were sub-optimal for binding and catalysis (Figure 6). While it would be difficult for Gua to fit in the tight active site of GDA, simple size exclusion could not explain the poor fitting of G, a molecule with a smaller size than Gua, into AtGSDA. The fact that 2′-O-mX could be efficiently bound by a non-canonical mode and acted upon suggested that the binding pocket of AtGSDA still has room for alternative binding possibilities. Actually, according to DoGSiteScorer, the 2′-O-mG-bound AtGSDA had a slightly larger pocket than the Xan-bound AtGSDA, suggesting the moderate plasticity of the binding pocket of the enzyme (Figure 7f and Figure S2). Therefore, the failure for G to bind to AtGSDA was probably due to loose interactions between the two. The former lacked the contacts with backbone of Asp140, Cys107-Glu108, due to the loss of the sugar moiety. In addition, the enzyme relies on the flexible C-tails to ensure a tightly binding pocket, which may “slip” away occasionally, defeating the purpose.

## 4. Materials and Methods

### 4.1. Cloning, Expression and Protein Purification

The full-length and the SF form of the gene (consisting of the Ser29-Tyr185 fragment) were inserted into the pET-21b (+) and the pET-28a (+) vectors, respectively, as described by Jia et al. [10]. All the mutations were generated using the QuikChange method (Stratagene). The expression and purification of all constructs followed the protocol as described previously [10]. The *Escherichia coli* GDA expression and purification followed the protocol as described by Sheck et al. [18].

### 4.2. Crystallization and Structure Determination

For the cocrystallization of the complexes, AtGSDA-SF variants were mixed with ligands at a molar ratio of 1:10, with the final concentration of the protein at 8.0 mg/mL. All of the cocrystals were obtained under the identical condition to that of the apo-protein [10]. The structures were solved by the molecular replacement method using Phaser [19] with the coordinates of apo-AtGSDA (PDB 7DBF) as the search model [10]. All the data collection and structure refinement statistics were summarized in Supplementary Table S1. The structural figures were produced with PyMOL (www.pymol.org) (accessed on 1 November 2022).

### 4.3. Thermal Shift Analysis

The parameters using in the thermal cycler program were described previously [10]. The assays were conducted in triplicates for all the mutants and the control.

### 4.4. Mass Spectrometry

The deamination reactions were conducted as described previously with small modifications [10]. Briefly, the enzyme (or mutants) and the substrate were incubated at a molar ratio of 1:100 for indicated periods, in the presence of 50 mM sodium bicarbonate in neutral condition. The reaction mix was taken out individually, inactivated by heating at 95 °C for 10 min and diluted threefold by dd H_2_O. Then it was injected into the high-resolution Orbitrap Fusion Lumos mass spectrometer (Thermo Fisher) and the resulting products were detected by either the positive or negative detection modes.

## 5. Conclusions

GDA and ADA have long been considered attractive drug targets for anticancer and antibacterial therapies. Here, we systematically explored the binding patterns of many potential competitive inhibitors of GSDA. Due to the considerable similarities in structure and catalytic pathways of these purine-metabolic enzymes, our efforts in novel substrate/inhibitor identification and subsequent structural characterization may provide an in-depth understanding of the paralogous enzymes. Additionally, the relatively large and plastic binding pocket of AtGSDA may provide more druggability than GDA and other related enzymes.

## Data Availability

Atomic coordinates and structure factors for the reported crystal structures have been deposited with the Protein Data bank under accession number 7DCW, 7DCB, 7DGC, 7DH1, 7DM6, 7DM5, 7DQN and 7W1Q.

## Supplementary Materials

The following supporting information can be downloaded at: https://www.mdpi.com/article/10.3390/ijms232314976/s1.
