# Supplementary material for: Substrate Specificity of GSDA Revealed by Cocrystal Structures and Binding Studies
Source: Int J Mol Sci. 2022 Nov 29;23(23):14976. doi: 10.3390/ijms232314976 (PMC9739398; doi:10.3390/ijms232314976)
Supplement: Supplementary file 1 [file ijms-23-14976-s001.zip › ijms-2003352-supplementary.pdf]

International Journal of
Molecular Sciences

MDPI

Supplementary Information

Substrate Specificity of GSDA Revealed by Cocrystal Structures and Binding Studies

Qian Jia, Jinbing Zhang, Hui Zeng, Jing Tang, Nan Xiao, Shangfang Gao, Huanxi Li and Wei Xie *

MOE Key Laboratory of Gene Function and Regulation, School of Life Sciences, Sun Yat-sen University, Guangzhou 510006, China

* Correspondence: xiewei6@mail.sysu.edu.cn

This PDF file includes: Figures S1-S2 and Table S1

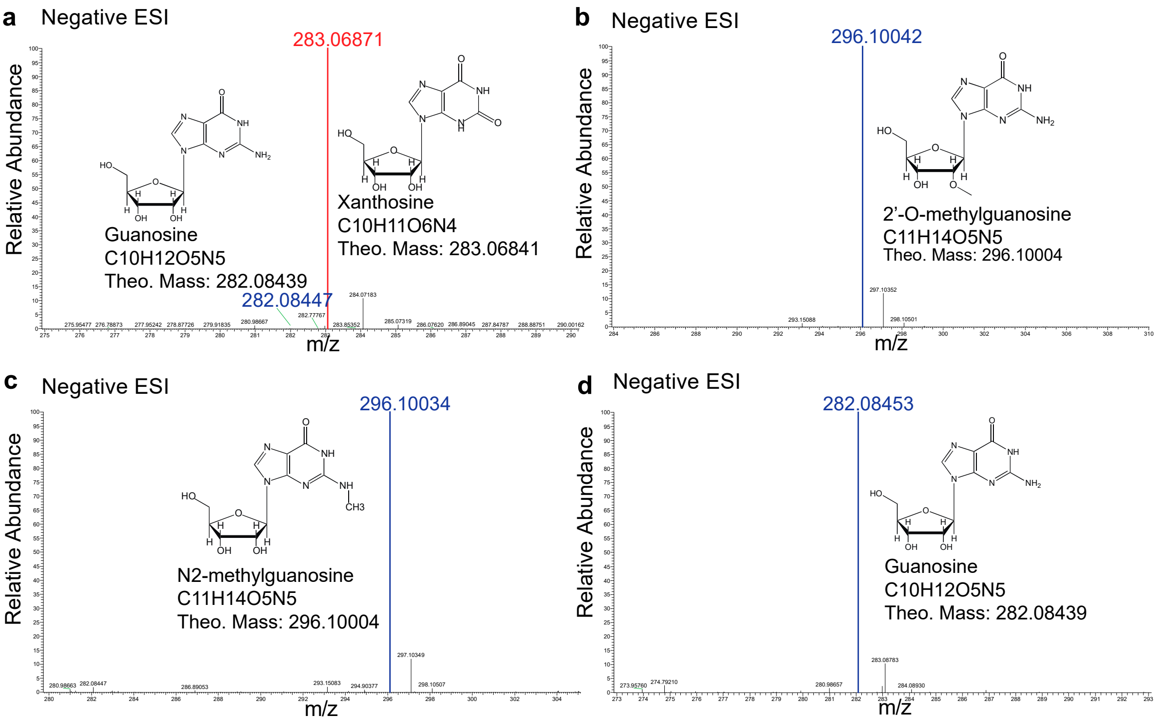

Figure S1. The deamination activity tests of AtGSDA and variants, analyzed by the Orbitrap high-resolution mass spectrometry. (**a**) The 2-hr deamination product by WT-AtGSDA incubation with Gua. (**b**-**d**) The 24-hr deamination product by AtGSDA/E82Q incubation with 2'-O-mG (**b**), with N^2-mG (**c**), and with Gua (**d**), respectively. The blue and red lines indicated the substrates and the products, respectively.

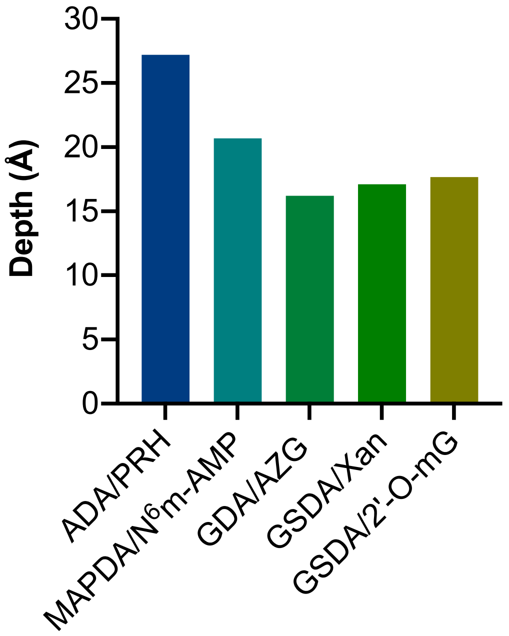

Figure S2. The depths of the substrate-binding pockets of several purine-metabolic enzymes. PRH: 6-hydroxy-1,6-dihydropurine riboside, N^6m-AMP: N^6-methyl-AMP; AZG: 8-azaguanine; Xan: xanthosine; 2'-O-mG: 2'-O-methylguanosine.

Table S1. Data collection and refinement statistics.

	WT-Ado (7DCW)	WT-Ins (7DCB)	WT-2'-O-mG (7DGC)
**Data collection**			
Space group	$P6_1$	$P6_1$	$P6_1$
Cell dimensions			
a, *b*, *c* (Å)	119.08, 119.08, 39.69	117.98, 117.98, 37.74	119.05, 119.05, 39.81
α, β, γ (°)	90, 90, 120	90, 90, 120	90, 90, 120
Resolution (Å)	21.63-2.30 (2.42-2.30)*	50-2.00 (2.07-2.00)*	50-2.10 (2.18-2.10)*
R_{merge}	0.24 (0.93)	0.22 (0.76)	0.24 (0.90)
$I / \sigma I$	10.5 (2.6)	19.2 (2.5)	19.1 (2.1)
Completeness (%)	99.9 (100)	99.5 (97.5)	99.9 (98.8)
Multiplicity	8.3 (6.8)	18.6 (13.8)	16.9 (10.0)
**Refinement**			
Resolution (Å)	21.64-2.30 (2.48-2.30)	34.06-2.00 (2.10-2.00)	31.51-2.11 (2.22-2.11)
No. reflections	14606	20493	18950
R_{work} / R_{free}	17.8/22.1	16.5/19.8	18.3/21.9
No. atoms			
Protein	2358	2359	2373
Ligand/ion	2 (Zn^{2+}), 38 (ADN)	2 (Zn^{2+}), 38 (NOS)	2 (Zn^{2+}), 42 (M2X), 1(Na^+)
Water	159	132	133
B-factors			
Protein	22.8	33.9	36.6
Ligand/ion	15.6 (Zn^{2+}), 20.5 (ADN)	25.7 (Zn^{2+}), 34.3 (NOS)	27.8 (Zn^{2+}), 42.0 (M2X), 52.8 (Na^+)
Water	24.9	38.8	40.5
R.m.s. deviations			
Bond lengths (Å)	0.003	0.004	0.002
Bond angles (°)	0.58	0.68	0.48
Ramachandran favored (%)	96.8	97.1	97.7
Outliers (%)	0.32	0.00	0.00

	WT-N^2-mG (7DH1)	WT-isoG (7DM6)	E82Q-2'-O-mG (7W1Q)
**Data collection**			
Space group	$P6_1$	$P6_1$	$P6_1$
Cell dimensions			
a, *b*, *c* (Å)	118.78, 118.78, 39.54	118.79, 118.79, 39.63	119.24, 119.24, 39.01
α, β, γ (°)	90, 90, 120	90, 90, 120	90, 90, 120
Resolution (Å)	50-1.92 (1.92-1.85)*	23.77-2.05 (2.16-2.05)*	23.69-2.30 (2.42-2.30)*
R_{merge}	0.12 (0.74)	0.11 (0.38)	0.52 (1.94)
$I / \sigma I$	28 (2.3)	17.6 (6.2)	15.0 (1.1)
Completeness (%)	99.6 (95.6)	99.9 (100)	99.9 (100)
Multiplicity	19.3 (16.3)	9.4 (9.7)	9.1 (7.5)
**Refinement**			
Resolution (Å)	31.35-1.85 (1.92-1.85)	23.77-2.05 (2.16-2.05)	23.69-2.30 (2.48-2.30)
No. reflections	27450	20365	14356
R_{work} / R_{free}	16.3/19.9	17.2/20.0	21.1/23.8
No. atoms			
Protein	2378	2368	2376
Ligand/ion	2 (Zn^{2+}), 40 (4UO)	2 (Zn^{2+}), 40 (ISG)	2 (Zn^{2+}), 21 (2MG)
Water	196	228	128
B-factors			
Protein	32.6	19.7	27.9
Ligand/ion	24.4 (Zn^{2+}), 31.5 (4UO)	11.6 (Zn^{2+}), 18.8 (ISG)	19.0 (Zn^{2+}), 35.9 (2MG)
Water	39.20	27.00	28.87
R.m.s. deviations			
Bond lengths (Å)	0.014	0.003	0.006
Bond angles (°)	1.25	0.53	0.88
Ramachandran favored (%)	97.4	97.1	97.7
Outliers (%)	0.00	0.32	0.00

	WT-Gua (7DM5)	Y185F-Gua (7DQN)
**Data collection**		
Space group	$P6_1$	$P6_1$
Cell dimensions		
a, *b*, *c* (Å)	118.57, 118.57, 39.41	119.17, 119.17, 39.48
α, β, γ (°)	90, 90, 120	90, 90, 120
Resolution (Å)	23.56-2.20 (2.32-2.20)*	23.78-2.60 (2.74-2.60)*
R_{merge}	0.11 (0.32)	0.16 (0.61)
$I / \sigma I$	15.1 (5.9)	12.1 (3.7)
Completeness (%)	99.9 (100)	99.9 (100)
Multiplicity	6.9 (7.1)	6.9 (7.1)
**Refinement**		
Resolution (Å)	23.56-2.20 (2.34-2.20)	23.78-2.60 (2.98-2.60)
No. reflections	16349	10089
R_{work} / R_{free}	16.7/22.2	16.6/22.1
No. atoms		
Protein	2390	2357
Ligand/ion	2 (Zn^{2+}), 40 (4UO)	2 (Zn^{2+}), 20 (GMP)
Water	197	92
B-factors		
Protein	20.9	27.6
Ligand/ion	12.5 (Zn^{2+}), 20.1 (4UO)	21.4 (Zn^{2+}), 31.5 (GMP)
Water	26.1	28.0
R.m.s. deviations		
Bond lengths (Å)	0.003	0.003
Bond angles (°)	0.55	0.56
Ramachandran favored (%)	97.4	97.7
Outliers (%)	0.00	0.00

*Values in parentheses are for highest-resolution shell. Each dataset was collected from a single crystal. Acronyms: Gua (GMP): guanosine; Xan (4UO): xanthosine; Ado (ADN): adenosine; Ins (NOS): inosine; 2'-O-mG (2MG): 2'-O-methylguanosine; M2X: 2'-O-methylxanthosine; N^2-mG: N^2-methylguanosine; isoG (ISG): isoguanosine. The codes in the parentheses were the three-letter designations for the ligands used for the PDB database.
